# The Reduced Oligomerization of MAVS Mediated by ROS Enhances the Cellular Radioresistance

**DOI:** 10.1155/2020/2167129

**Published:** 2020-03-03

**Authors:** Yarong Du, Dong Pan, Rong Jia, Yaxiong Chen, Cong Jia, Jufang Wang, Burong Hu

**Affiliations:** ^1^Key Laboratory of Space Radiobiology of Gansu Province & CAS Key Laboratory of Heavy Ion Radiation Biology and Medicine, Institute of Modern Physics, Chinese Academy of Sciences, Lanzhou 730000, China; ^2^College of Life Science, Northwest Normal University, Lanzhou 730070, China; ^3^School of Nuclear Science and Technology, University of Chinese Academy of Sciences, Beijing 100039, China

## Abstract

Although the mitochondrial antiviral signaling protein (MAVS), located in the mitochondrial outmembrane, is believed to be a signaling adaptor with antiviral feature firstly, it has been shown that suppression of MAVS enhanced radioresistance. The mechanisms underlying this radioresistance remain unclear. Our current study demonstrated that knockdown of MAVS alleviated the radiation-induced mitochondrial dysfunction (mitochondrial membrane potential disruption and ATP production), downregulated the expressions of proapoptotic proteins, and reduced the generation of ROS in cells after irradiation. Furthermore, inhibition of mitochondrial ROS by the mitochondria-targeted antioxidant MitoQ reduced amounts of oligomerized MAVS after irradiation compared with the control group and also prevented the incidence of MN and increased the survival fraction of normal A549 cells after irradiation. To our knowledge, it is the first report to indicate that MAVS, an innate immune signaling molecule, is involved in radiation response via its oligomerization mediated by radiation-induced ROS, which may be a potential target for the precise radiotherapy or radioprotection.

## 1. Introduction

Mitochondrial antiviral signaling protein (MAVS), a signaling adaptor with antiviral feature in the mitochondrial membrane, is critical for host defenses against viral infection. The homeotypic interaction between the domains of a caspase recruitment domain (CARD) of MAVS and the CARD of RIG-I forms protein aggregates on the surface of the mitochondria that can further activate MAVS proteins to form functional clusters to propagate antiviral innate immune response [1]. These high molecular weight MAVS complexes then recruit the IKK and TBK1/IKKi complexes to induce transcriptional expression of type I interferon (IFN) by promoting the nuclear translocation of the NF-*κ*B and IRF3/7 transcription factors, respectively, and thus elicit the innate antiviral response (F. [2, 3]). Radiation-mediated NF-*κ*B activation can directly induce expression of several proinflammatory cytokines including TNF, IL-1*α*, and IL-1*β* [4–6]. Thereby, radiotherapy can also activate innate and adaptive immune responses against tumors [7–10]. MAVS is involved in IFN-beta and IFN-stimulated gene expression in the response to ionizing radiation (IR). It was reported that physiologic responses to radio-/chemotherapy converge on an antiviral program in recruitment of the RLR pathway by a sncRNA- (small nuclear RNAs U1 and U2-) dependent activation of RIG-I which commences cytotoxic IFN signaling, and suppression of MAVS conferred radioresistance in normal and cancer cells [11]. However, the underlying mechanisms on MAVS suppression resulting in radioresistance remain poorly understood.

Mitochondria are involved in many important cell processes including cell respiration, reactive oxygen species (ROS) production, and apoptosis induction. Accumulating data indicate that IR damages the mitochondrial structure (mass, morphology) and induces the dysfunctions of mitochondria in cells, such as the disorder of cellular respiration, changes of calcium balance and membrane potential, and elevation of ROS level, which result in the radiosensitivity [12–14]. MAVS is predominantly localized and executes its functions at the outer membrane of the mitochondria. It is not clear whether the change of MAVS expression influences the mitochondrial functions responding to IR and results in the radiosensitivity. Hou et al. reported that increased cellular ROS promoted MAVS forming functional prion-like aggregates to activate and propagate antiviral innate immune response [1, 15]. Conversely, repression of mitochondrial ROS (mtROS) production by cytochrome C oxidase complex subunit 5 inhibits MAVS aggregation and the downstream NF-*κ*B and IRF3/7 signaling pathway [16]. IR can induce the generation of ROS. Whether and how did the increase of radiosenstivity partially via the activation of MAVS aggregation by radiation induce ROS?

In the present work, we attempted to investigate the mechanisms of radioresistance in cells after MAVS suppression. Our results demonstrated that suppression of MAVS alleviated the radiation-induced mitochondrial dysfunction and reduced the generation of ROS, compared to the normal cells. We also observed that more large aggregates of MAVS formed after IR and these MAVS aggregates lead to a gain of function in activating downstream factors. Radiation-induced MAVS oligomerization was inhibited by the addition of MitoQ, a mitochondria-targeted antioxidant, and decreased the radiosensitivity. Our results suggest a mechanism that MAVS oligomerization induced by radiation plays a role in the induction of radiosenstivity.

## 2. Materials and Methods

### 2.1. Cell Culture

A549 and BEAS-2B cells were obtained from Chinese Center for Disease Control and Prevention and cultured in RPMI 1640 medium (Gibco, USA) supplemented with 10% fetal bovine serum (Hyclone, USA) and 1% penicillin/streptomycin (Amresco, USA). HepG2 and MCF7 cells were purchased from Shanghai Cell Bank. Cells were cultured in DMEM medium (Gibco, USA) supplemented with 10% fetal bovine serum (Hyclone, USA) and 1% penicillin/streptomycin (Amresco, USA) at 37°C in a humidified atmosphere containing 5% CO_2_. MitoQ was purchased from MedChemExpress.

### 2.2. Radiation

For X-ray irradiation, an X-ray facility (target: W, Faxitron Bioptics, USA) was used. The dose rate was ~0.78 Gy/min.

### 2.3. Gene Transfection

A549 cells were transfected with siRNA oligos at a final concentration of 50 nM. siRNA that targets MAVS and its negative control were purchased from RiboBio (Guangzhou, China). MAVS siRNAs (sense: 5′CCACCUUGAUGCCUGUGAATT-3′, antisense: 5′ UUCACAGGCAUCAAGGUGGTT-3′) were constructed as described [17]. Cells on the day before transfection were at a 70% of confluence and then, transfection was performed with Lipofectamine 2000 (Invitrogen, USA) according to the manufacturer's instructions. The medium was exchanged for new culture medium 6 h posttransfection. The cells at 48 h after transfection were used in the following experiments.

### 2.4. Micronuclei (MN) Scoring

MN formation analysis is another generally used biological endpoint for the study of radiation effect. In brief, the cells in the slides were irradiated and continuously cultured for 48 h. The cells were fixed with Carnoy's solution for 20 min at room temperature and then stained with 20 *μ*L of acridine orange in an aqueous solution (10 *μ*g/mL). The cellular images were taken under the fluorescence microscope (Axio Imager. Z2) at 20x magnification, and 500 cells at least for each sample were scored, and cells with MN were calculated. Each experiment was repeated three times independently at least.

### 2.5. Colony Formation Assay

Briefly, the cells were trypsinized immediately after irradiation treatment and resuspended in RPMI 1640 medium with 10% FBS. A549 or BEAS-2B cells in appropriate amount were plated into each 60 mm dish to produce colonies. After being cultured for 14 days, the medium were removed and cells were stained with 0.5% crystal violet for 30 min. Colonies with more than 50 cells were manually counted. Plating efficiencies (PE) were calculated as follows: numbers of colonies formed/numbers of cells plated. Surviving fractions were calculated as follows: PE (irradiated)/PE (unirradiated).

### 2.6. Western Blot Assay

Cells were lysed in RIPA buffer (Beyotime, China) with Protease Inhibitor Cocktail Tablets (Roche, Switzerland). The protein concentration was quantified using a BCA protein assay kit (Thermo Scientific, USA). Equal amounts of protein were denatured with loading buffer (Beyotime, China) at 100°C for 10 min, subjected to 12% SDS-PAGE, and then blotted to a methanol-activated PVDF membrane (Millipore, USA). After blocked with 5% bovine serum albumin (ABCONE, China) in tris-buffered saline (TBS) for 1 h at room temperature, the membranes were respectively incubated overnight with the following antibodies at 4°C: MAVS (1 : 1000, Abcam, USA); pho-IRF3 (1 : 1000, Abcam, USA); caspase-3 (1 : 1000, Cell Signaling Technology, USA); IFN-I, IRF3, cyto C, Bax, and Bcl2 (1 : 1000, Affinity Biosciences, USA); and beta-actin (1 : 1000, ZSGB-BIO, China). After being washed for three times with TBS, the membranes were incubated with the horseradish peroxidase- (HRP-) labeled secondary antibody (1 : 2500, ZSGB-BIO, China) for 1 h at room temperature.

### 2.7. Mitochondrial Membrane Potential (MMP) Measurement

The status of the cellular mitochondrial membrane potential (*ΔΨ*m) was evaluated using JC-1 (BD Biosciences) and was quantified by the microplate reader (Tecan Infinite 200 M) [18]. Briefly, after being transfected with MAVS siRNA oligos for 48 h and followed by 2 Gy X-ray irradiation for 24 h, the cells (~1 × 10^6^ cells/mL) were washed once with PBS and harvested into a centrifuge tube. Cells were resuspended in 500 *μ*L JC-1 working solution, incubated at 37°C for 15 min, then washed twice with 1x assay buffer, and resuspended each cell pellet in 500 *μ*L of 1x assay buffer. Microplate reader and immunofluorescence microscopy (Axio Imager Z2) analysis at 10x magnification were performed after staining.

### 2.8. Cellular ATP Content

The intracellular ATP content was evaluated by measuring the luminescence with an ATP determination kit (Thermo Fisher, USA) according to the manufacturer's protocol. Briefly, the MAVS knockdown cells were irradiated by 0 and 2 Gy X-rays and then incubated for 1, 12, and 24 h. The cells were lysed in RIPA buffer on ice (Beyotime, China), and then, the supernatants were collected into 1.5 mL tubes. The chemiluminescence from each well was measured with an Infinite 200 Microplate Reader (Tecan, Switzerland) set at 25°C. The standard curve for a series of ATP concentrations was confirmed. ATP concentrations of sample were calculated from the standard curve. To eliminate errors caused by differences in sample content, we use a Bradford protein assay kit to normalize protein quantity. The amount of ATP was expressed as mmol/g of protein.

### 2.9. Apoptosis Assays

At the end of all interventions, A549 (>1 × 10^5^) cells were collected and stained with Annexin V-FITC/propidium iodide (PI) Kit (BD, USA) according to the manufacturer's protocol. The apoptosis of A549 was measured by a flow cytometer FlowSight (Amnis, USA).

### 2.10. ROS Level Measurement

The fluorescent probe, 2′7′-dichlorofluorescin (DCFH-DA, Sigma, USA), was employed to quantify the level of ROS as described [19]. The medium of the cells to be detected was removed at the designed time points. Cells were washed once with 1x PBS solution and then stained with 10 *μ*M DCFH-DA for 30 min at 37°C in the dark. The fluorescence images were taken under the fluorescence microscopy (Axio Imager Z2), and the relative fluorescence intensity for each treatment was quantified by means of ImageJ software.

### 2.11. Semidenaturing Detergent Agarose Gel Electrophoresis (SDD-AGE)

SDD-AGE was performed according to a published protocol with minor modifications [20]. Briefly, equal amounts of the whole cell lysate were resuspended in one-third volume of 4x loading buffer consisting of 20% glycerol, 8% SDS, and 0.01% bromophenol blue in 2x TAE. Samples were incubated for 5 min at room temperature and loaded onto a vertical 1.5% agarose gel containing a final concentration of 0.1% SDS. Migration was performed for 35 min with a constant voltage of 100 V at 4°C. Proteins were transferred to PVDF membranes using a liquid transfer system in preparation for western blotting analysis. In order to confirm the consistency of the sample amounts, save half of the sample volume and boil it for western blotting analysis.

### 2.12. Level of Cytokine Measurement by ELISA Analysis

The cellular media of each treated group were collected at the indicated time points, and the levels of IL6, TNF-*α*, and IL-1*β* in the media were measured using ELISA kits (eBioscience) following the manufacturer's instructions. The fold changes of IL-6, TNF-*α*, and IL-1*β* levels were analyzed among different treatment group.

### 2.13. Statistical Analysis

The data were presented as mean ± SD of three independent experiments at least. The statistical significance (*P* value) was determined using Student's *t*-test for single comparisons and analysis of variance (ANOVA) for statistical comparison between different groups. If the *P* value < 0.05 was considered statistically significant between two sample comparison.

## 3. Results

### 3.1. MAVS Is Involved in Radiation Response

**Figure 1:**
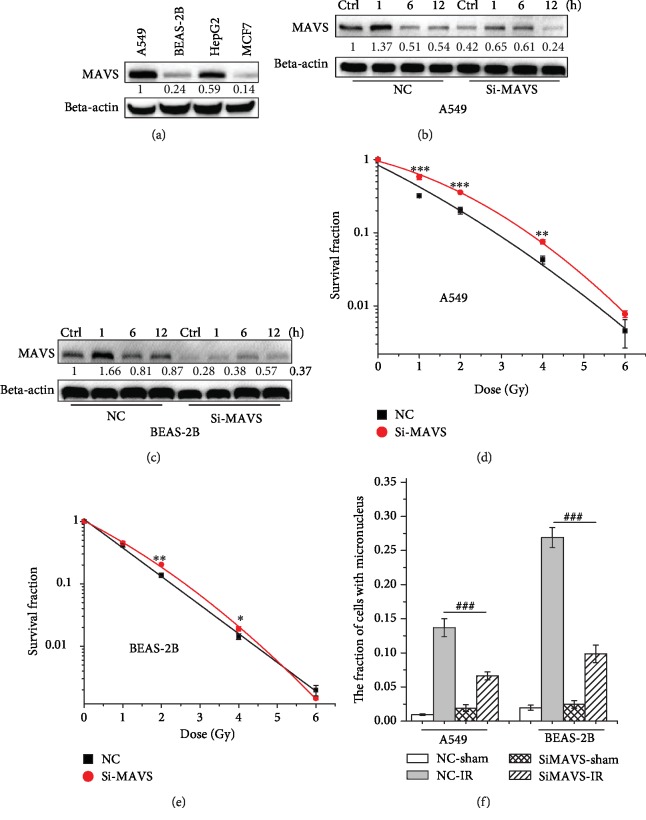
MAVS knockdown is resistant to radiation response. (a) The comparison of MAVS expression among A549, BEAS-2B, HepG2, and MCF7 cells. The expressions of MAVS at the indicated time points after 2 Gy X-rays in negative vector or MAVS-silenced A549 cells (b) and BEAS-2B cells (c) by western blot assay. Survival in A549 cells (d) and BEAS-2B cells (e) transfected with siRNA-MAVS or negative vector and then exposed to 0, 1, 2, 4, or 6 Gy X-rays measured by colony formation assay. (f) The fraction analysis of MN in negative vector and MAVS silenced in A549 and BEAS-2B cells after 2 Gy X-ray irradiation. Data are presented as mean ± SD. ^∗^*P* < 0.05, ^∗∗^*P* < 0.01, and ^∗∗∗^*P* < 0.001 vs. the control group; ^###^*P* < 0.001 vs. the irradiation group.

The levels of MAVS expression in different cell lines (A549, BEAS-2B, HepG2, and MCF7) were also analyzed.  Figure 1(a) shows that the expressions of MAVS in BEAS-2B and MCF7 cells were lower. The expressions of MAVS were upregulated in both A549 (Figure 1(b)) and BEAS-2B (Figure 1(c)) cells at 1 h after X-ray radiation and then decreased. After being transfected with siRNA, the expression of MAVS in two cell lines was silenced and the upregulations of MAVS expressions induced by radiation were suppressed effectively, compared to the normal irradiated cells. Further, colony formation assays revealed that knockdown of MAVS gene increased the survival fraction of A549 (Figure 1(d)) and BEAS-2B (Figure 1(e)) after irradiation, compared to the normal control (NC) irradiation group. Consistently, knockdown of MAVS gene greatly diminished the incidence of MN in A549 and BEAS-2B cells after irradiation, compared to those NC cells after radiation (Figure 1(f)). These results confirm that MAVS responds to radiation and knockdown of MAVS increases the radioresistance.

### 3.2. Knockdown of MAVS Attenuates Radiation-Triggered MMP Disruption

**Figure 2:**
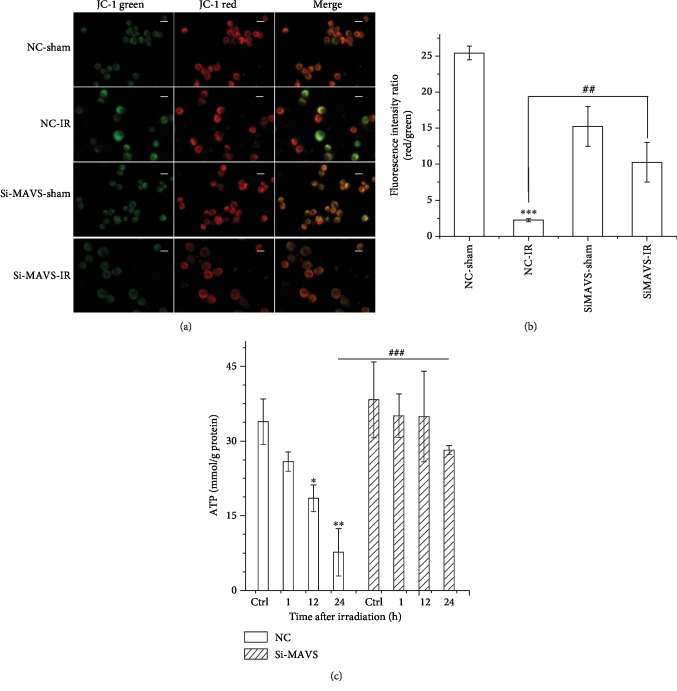
The impact of MAVS silencing combined with irradiation on the MMP and the mitochondrial ATP production of cells. The MMP of negative vector or MAVS-silenced cells at 24 h after 2 Gy X-ray irradiation was assessed by fluorescence microscope (a) and quantified by the microplate reader (b). Scale bar represents 10 *μ*m. (c) The content of ATP was detected by ATP assay kit in negative vector and MAVS-silenced cells after irradiation. Data are presented as mean ± SD. ^∗∗∗^*P* < 0.001 vs. the control group; ^##^*P* < 0.01 vs. the irradiation group. Red fluorescent represents intact MMP, and green fluorescent represents dissipation of MMP.

MMP is a key indicator of mitochondrial function and activity because it reflects the process of elector transport and oxidative phosphorylation and is the driving force for mitochondrial ATP synthesis. Consequently, mitochondrial depolarization exhibited decreased red fluorescence and enhanced green fluorescence, and a collapse in the *ΔΨ*m is indicated by a reduction in the red/green fluorescence intensity ratio [21]. To investigate the effects of MAVS suppression on mitochondrial functions after irradiation, we carried out the measurement of MMP.  Figure 2(a) shows that the states of JC-1 monomers (green color cell) and JC-1 aggregates (red color cell) in normal or MAVS knockdown cell lines after irradiation.  Figure 2(b) shows the knockdown of MAVS prevented the radiation-induced decrease in the red-to-green fluorescence intensity ratio of JC-1 staining measured by the microplate reader. After irradiation, the fluorescence ratio of JC-1 aggregates dramatically decreased in the irradiated normal A549 cell lines at 24 h time point, while these ratios did not change obviously in the irradiated cells with MAVS knockdown, compared to their control cells. These results suggest that knockdown of MAVS attenuates radiation-triggered MMP disruption.

### 3.3. Knockdown of MAVS Alleviates the Reduction of ATP Production of Cells after Irradiation

Mitochondrial ATP content is a classic indicator of mitochondrial respiration function. We wanted to know whether the knockdown of MAVS influenced the ATP production and what would happen in the MAVS knockdown cells after irradiation. The levels of cellular ATP in the cells were measured using a luciferin/luciferase kit. As shown in Figure 2(c), the cellular ATP content remained unchanged in both normal and knockdown cells at 1 h after irradiation. Interestingly, the content of ATP in the normal irradiated cells at 24 h post-irradiation dramatically decreased, compared to that in MAVS knockdown cells. These data suggest that irradiation stimulation decreases the process of mitochondrial energy metabolism of cell and MAVS knockdown partially recovers the production of mitochondrial energy under the condition of stress stimulation.

### 3.4. Knockdown of MAVS Downregulates the Expressions of Apoptosis-Related Protein

**Figure 3:**
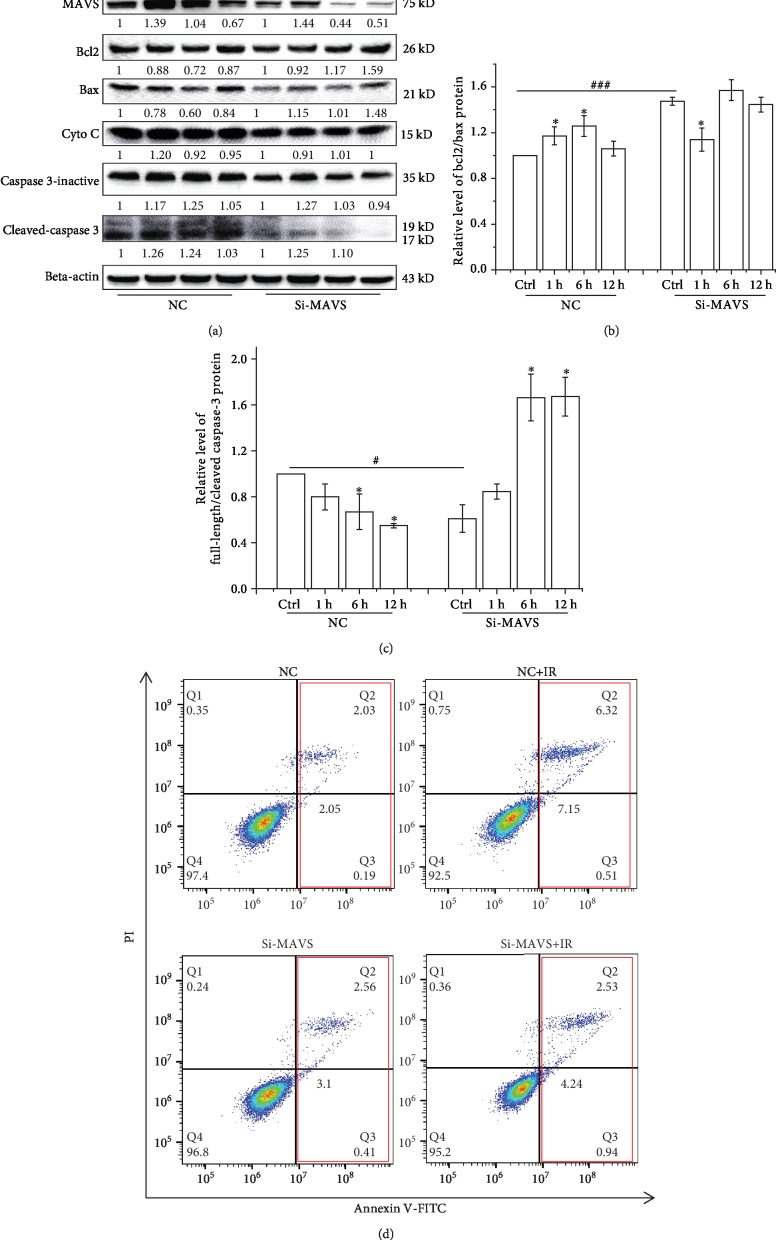
Effects of MAVS ectopic expression on cell apoptosis and the changes in regulatory proteins induced in negative vector and MAVS-silenced cells after irradiation. (a) The expression of apoptosis-related proteins was assessed and quantified in negative vector or MAVS-silenced A549 cells after 2 Gy X-ray irradiation by western blotting. (b) Value of Bcl2/Bax protein expression quantified using ImageJ software was presented. (c) Value of full length/cleaved caspase-3 protein expression quantified using ImageJ software was presented. (d) Apoptosis was quantified by the combined staining of Annexin V and PI, and fluorescence was analyzed using flow cytometry in negative vector and MAVS-silenced A549 cells after irradiation. Each data point represents the mean of three separate experiments. Values are presented as mean ± SD. ^∗^*P* < 0.05, ^∗∗^*P* < 0.01, and ^∗∗∗^*P* < 0.001 vs. the control group; ^#^*P* < 0.05, ^##^*P* < 0.01, and ^###^*P* < 0.001 vs. the irradiation group.

Mitochondrial damage facilitates cytochrome C (cyto C) release from mitochondria into the cytoplasm and activates Bcl-2 family proteins, which leads to activation of the caspase cascade (apoptotic markers) and cellular apoptosis [22]. Our above results showed that knockdown of MAVS led to the alleviation of radiation-triggered MMP disruption. Thus, the expression levels of mitochondrial apoptosis-associated proteins, such as cyto C, caspase-3, Bax, and Bcl-2, were determined by western blot to deduce the role of MAVS suppression in the radiation-induced cell apoptosis.  Figure 3(a) shows that MAVS knockdown suppressed the expression of cyto C, caspase-3-inactive (total caspase-3 protein), cleaved-caspase-3, and Bax. Cyto C expression increased in normal A549 cells at 1 h after irradiation, while kept relatively constant in MAVS knockdown cells after irradiation. The total caspase-3 expression increased at 1-6 h and then decreased at 12 h in the normal A549 cell after irradiation, while only increased at 1 h and then decreased at 6 h in the irradiated MAVS knockdown cells. However, the expression of activated caspase-3 (cleaved caspase-3) significantly increased in normal cells at 1-6 h after irradiation, while there is no obvious change in the MAVS knockdown A549 cells. Compared to the respective control, the expressions of activated caspase-3 were blunted in the irradiated MAVS knockdown cells. Overexpression of Bcl2 promotes cellular survival, and overexpression of Bax enhances the cellular apoptosis. Thus, the higher the ratio of Bcl2/Bax, the more cellular survival it reflects [23]. In comparison to the normal A549, the ratio of Bcl2/Bax is slightly higher in MAVS knockdown A549 cells at 6 and 12 h after irradiation (Figure 3(b)). These results indicate that knockdown of MAVS suppresses the expressions of apoptosis-associated proteins and protect the cells from the radiation-induced death. Meanwhile, our results from flow cytometry confirmed that knockdown of MAVS inhibited cell apoptosis after X-ray radiation (Figure 3(c)).

### 3.5. Knockdown of MAVS Inhibits the Generation of Radiation-Induced ROS

**Figure 4:**
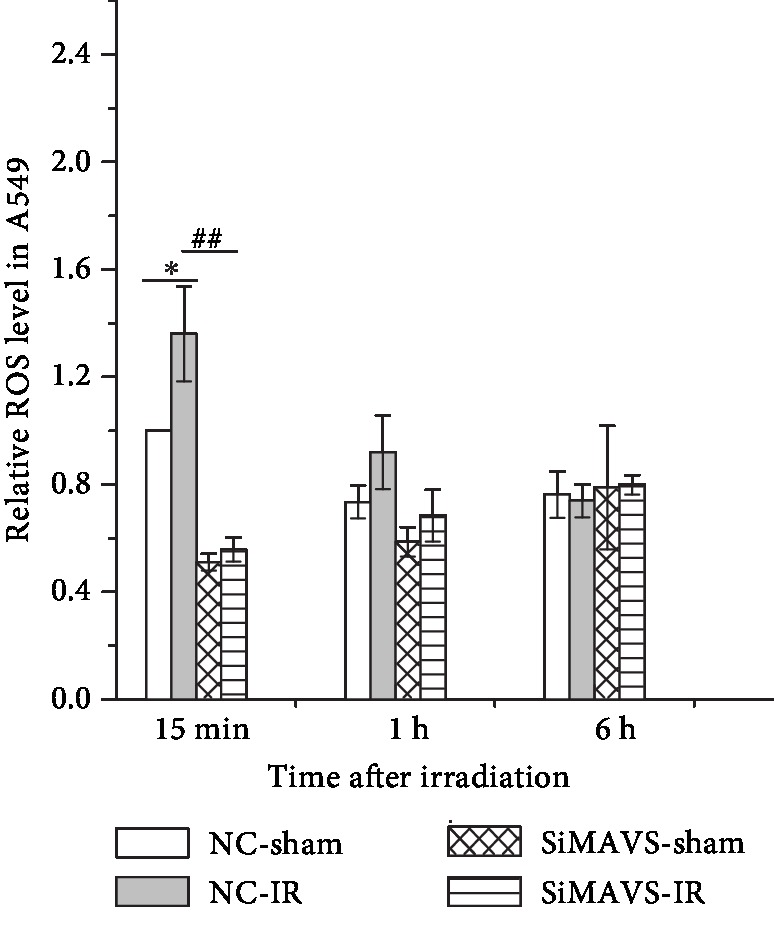
The relative levels of ROS induced by 2 Gy X-ray radiation in negative vector and MAVS-silenced A549 cells. Data are presented as mean ± SD. ^∗^*P* < 0.05 vs. the control group; ^##^*P* < 0.01 vs. the irradiation group.

Generation of higher level of ROS in cells is related to cell apoptosis [24]. To determine the relationship between the knockdown of MAVS and the level of ROS after irradiation, we measured the ROS level in cells at 15 min, 1, and 6 h after exposed to 2 Gy X-rays.  Figure 4 shows that the ROS level increased in normal A549 cells at 15 min after irradiation, while it remained relatively constant in the MAVS knockdown cells after irradiation. Compared to the irradiated MAVS knockdown A549 cells, the ROS level in the normal cells increased by 3.0-fold at 15 min after irradiation. There were no big differences between normal and MAVS knockdown A549 cells at 1 and 6 h after irradiation.

### 3.6. The Mitochondria-Targeted Antioxidant MitoQ Reduces MAVS Oligomerization

**Figure 5:**
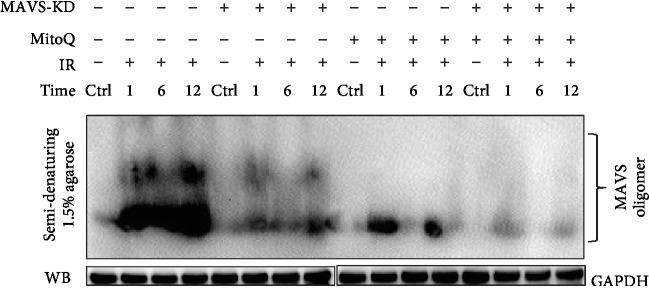
MAVS oligomerization was detected in negative vector or MAVS-silenced A549 cells at the indicated times after X-ray radiation by nonreducing gels and western blotting analysis. To define the role of ROS in MAVS oligomerization, MitoQ was used as scavenging mitochondria ROS agent.

Study demonstrated that increased cellular ROS promoted MAVS aggregates to activate the downstream signaling response [1]. To evaluate the influence of radiation or radiation-induced ROS on MAVS oligomerization, we adapted a SDD-AGE system to detect MAVS aggregates after irradiation. MitoQ is a mitochondria-targeted antioxidant, which contains ubiquinol as the active antioxidant component [25]. Cells pretreated with 1 *μ*m MitoQ for 2 h dramatically reduced MAVS oligomerization, compared to the X-ray radiation alone group, which is at least a 75% of decrease in the amount of MAVS oligomers (Figure 5), while there is no increase of MAVS oligomers in MAVS knockdown A549 cells after irradiation. These results suggest that radiation-induced ROS activate the oligomerization of MAVS and the phenomenon will be reduced if the MAVS is suppressed.

### 3.7. The Pretreatment of MitoQ Reduces the Cellular Radiosensitivity

**Figure 6:**
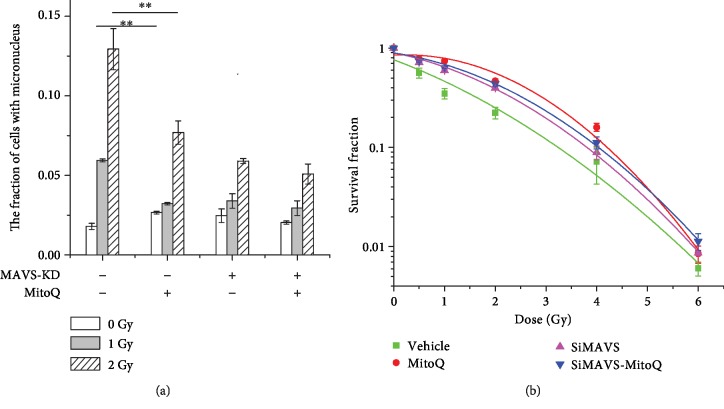
Biological effect changes in negative vector and MAVS-silenced A549 cells after being pretreated with MitoQ and X-ray irradiation. (a) The fraction analysis of MN in negative vector and MAVS-silenced A549 cells pretreated with MitoQ for 2 h and then radiated with 1 Gy and 2 Gy X-ray. (b) The survival analysis of negative vector and MAVS-silenced A549 cells pretreated with MitoQ after exposed to 0, 1, 2, 4, and 6 Gy X-rays. Data are presented as mean ± SD. ^∗^*P* < 0.05, ^∗∗^*P* < 0.01, and ^∗∗∗^*P* < 0.001 vs. the irradiation group.

Figure 6(a) shows that the incidence of MN was greatly diminished in A549 cells pretreated with MitoQ, compared to the knockdown of MAVS after 1 Gy or 2 Gy X-ray irradiation. Further, survival fraction of A549 cells pretreated with MitoQ and then irradiation was increased, compared to the irradiated cells alone (Figure 6(b)). MitoQ did not affect the biological effect of MAVS knockdown cell lines after irradiation.

### 3.8. Knockdown of MAVS Inhibits the Generation of IL6 and Expression of IFN

**Figure 7:**
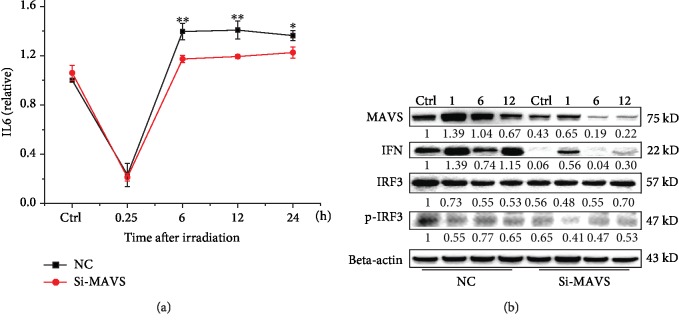
Associated immune factor was detected by ELISA assay (a) and western blotting (b) in negative vector and MAVS-silenced A549 cells at the indicated times after X-ray radiation. Data are presented as mean ± SD. ^∗^*P* < 0.05, ^∗∗^*P* < 0.01, and ^∗∗∗^*P* < 0.001 vs. the irradiation group.

Cytokines, such as IL6, TGF*α*, and IL1*β*, are the downstream of MAVS signaling pathway and involve in radiation induced sensitivity. Thus, we detected the relative level of these cytokines in normal and MAVS knockdown cells after irradiation. Irradiation stimulation reduced the level of IL6 at early time (15 min after) then induced the upregulation of level of IL6 at 6 h time point. Knockdown of MAVS suppressed the level of IL6 after irradiation, compared to the irradiated normal cells (Figure 7(a)). The levels of TGF*α* and IL1*β* were not obviously different between the normal and MAVS knockdown cells after irradiation (data not shown).  Figure 7(b) shows that knockdown of MAVS suppressed the expression of IFN and also inhibited the expression of total IRF3 (IFN regulatory factor-3) and pho-IRF3, suggesting that MAVS knockdown suppresses the inflammation signaling pathway and may contribute to the radioresistance.

## 4. Discussion

IR, because of its cell killing capability, has been utilized to therapy cancers, and radiotherapy is one of the three main therapies (surgery, chemotherapy, and radiotherapy) for many types of tumor [26]. Radiotherapy can increase innate and adaptive immune responses against tumors, and the type I IFN signaling pathway is involved in this process [27]. It was reported that MAVS is necessary for IFN-beta induction and interferon-stimulated gene expression in the response to IR and suppression of MAVS conferred radioresistance in normal and cancer cells [11]. However, the mechanisms underlying this process are largely unknown.

In the current study, we first verified the phenomenon of suppression of MAVS conferring radioresistance by means of clone survival and MN assay. Further, because MAVS is a protein which localized the outer membrane of mitochondrial, we attempted to investigate the influences of suppression of MAVS on mitochondrial functions to probe the potential mechanisms. Although we also observed a slight changes of MMP in MAVS-silenced cells, the cellular collapse of the *ΔΨ*m in the irradiated normal cells was dramatically more than that in the irradiated MAVS-silenced cells at 24 h after irradiation. The content of ATP in normal cells after irradiation dramatically decreased, but the decrease was relieved in the MAVS-silenced cells after irradiation. Mitochondrial dysfunction can lead to the release of Cyto C and cell apoptosis. Our results demonstrated that the expressions of apoptosis protein Cyto C and active caspase-3 in the irradiated MAVS knockdown cell line were lower and the ratio of Bcl2/Bax proteins was higher than that of the irradiated normal cells, indicating that apoptosis decreased in MAVS knockdown cells after irradiation. These data suggest that knockdown of MAVS not only inhibit the radiation-induced mitochondrial dysfunction but also block the transmission of cellular apoptosis signaling pathway, which results in the subsequent radioresistance and may also indicate one of mechanisms on radioresistance.

In general case, except for the direct damage to DNA and other cellular components, IR also instantaneously causes the formation of water radiolysis products that contain ROS (chemical process occurred in the extremely early stage of radiation). ROS are also suggested to be released from biological sources in irradiated cells [28]. In our study, we observed that generation of radiation-induced ROS in MAVS knockdown cells decreased by 3.0-fold at 15 min after irradiation. Buskiewicz et al. reported that chemically generated oxidative stress (using glucose oxidase) stimulated the formation of MAVS oligomers, which led to mitochondrial hyperpolarization and decreased ATP production and spare respiratory capacity [29]. MAVS oligomerization directly suppresses the function of mitochondrial complexes I to IV and trigger the deficiency of ATP and the effusion of Cyto C [30]. Based on their findings, we speculate that MAVS involves in radiosensitivity through its oligomerization mediated by ROS.

**Figure 8:**
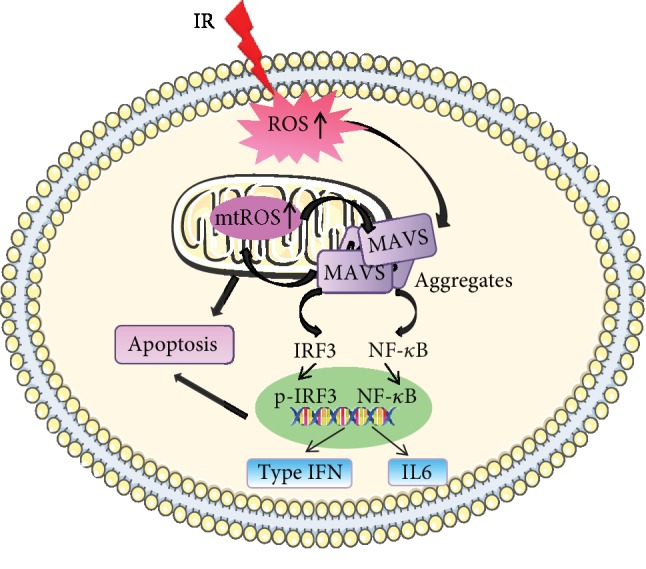
The speculated model of ROS-mediated MAVS oligomerization resulting in the radioresistance.

According to their measure method, we first observed that the irradiation increased the oligomerization of MAVS in A549, which was significantly inhibited by MitoQ pretreatment, and there was no increased oligomer of MAVS in the knockdown cell. MitoQ pretreatment also decreased the radiation-induced fractions of MN and increased the survival of normal A549 cells. Additionally, knockdown of MAVS also suppressed the expression of IRF3, type I IFNs, and almost eliminated pho-IRF3. Radiation stimulation induced the upregulation of IL6 level at 6 h after IR. Knockdown of MAVS suppressed the increase of the level of IL6 after IR. These results suggest that oligomerization of MAVS mediated by radiation-induced ROS form prion-like aggregates to induce the mitochondrial dysfunction and the activation NF-*κ*B and IFN-type I. Suppression of MAVS reduces MAVS oligomer, thus decreasing radiation-induced mitochondrial dysfunction and inhibiting the pathway of cell apoptosis and the release of part of proinflammation cytokines, as well as the generation of mtROS again, resulting in the radioresistance. These findings imply a model that ROS generation during initial chemical progress after irradiation could be an initial upstream signaling factor of MAVS responding to radiation, and then this kind of ROS-mediating initial MAVS oligomerization induces the increase of mtROS, which in turn induces more oligomers of MAVS and expand the radiation-induced damage (Figure 8). In order to confirm the conclusion, we also constructed the overexpression MAVS plasmid and transfected to A549 or BEAS-2B cell lines but unfortunately could not get stable over expression cell lines. From previous experiment results, we speculated the occurrence of mitochondrial dysfunction and apoptosis in MAVS overexpression, resulting to apoptosis or necrosis in the MAVS overexpression cell lines.

## 5. Conclusions

MAVS, an innate immune signaling molecule, is involved in radiation response via its oligomerization mediated by radiation-induced ROS. Knockdown of MAVS alleviates the radiation-induced mitochondrial dysfunction and decreases the expressions of proapoptotic proteins in cells after irradiation, which results in the radioresistance. Our study implies that MAVS may be a potential target for the precise radiotherapy or radioprotection.

## Data Availability

The data used to support the findings of this study are available from the corresponding authors upon request.
